# Development and validation of a machine learning scoring system based on multi-dimensional metabolic signatures for invasive examination triage in intestinal diseases

**DOI:** 10.3389/fgene.2026.1905165

**Published:** 2026-09-07

**Authors:** Jintao He, Jiaorong Chen, Yong Zhang, Yingbo Rao, Mengning He, Mingli Zhu

**Affiliations:** 1 School of Medical Technology and Information Engineering, Zhejiang Chinese Medical University, Hangzhou, Zhejiang, China; 2 The Fourth Affiliated Hospital, Zhejiang University School of Medicine, Yiwu, Zhejiang, China; 3 Department of Clinical Laboratory, The First Affiliated Hospital, Ningbo University, Ningbo, Zhejiang, China; 4 Open Laboratory, Hangzhou Xixi Hospital, Zhejiang Chinese Medical University, Hangzhou, Zhejiang, China

**Keywords:** endoscopic triage, inflammatory bowel disease, irritable bowel syndrome, machine learning, metabolic signatures, metabolism-related biomarkers

## Abstract

**Background:**

Inflammatory bowel disease (IBD) and irritable bowel syndrome (IBS) often present with overlapping gastrointestinal symptoms despite distinct pathophysiological mechanisms. Colonoscopy remains the diagnostic gold standard but is invasive, costly and frequently overutilized. Single biomarkers have limited diagnostic value because they do not fully reflect the complex metabolic and inflammatory alterations underlying intestinal diseases. We aimed to develop and validate an interpretable machine learning-based scoring system integrating multidimensional metabolism-related biomarkers for invasive examination triage.

**Methods:**

This retrospective single-center study included 729 participants (313 healthy controls, 210 IBS, 100 ulcerative colitis and 106 Crohn’s disease patients) enrolled between July 2021 and November 2025. Demographic, clinical, and metabolism-related laboratory biomarkers reflecting inflammatory metabolism, nutritional metabolism, hepatic metabolic function, and renal metabolic homeostasis were collected. Correlation network analysis, least absolute shrinkage and selection operator (LASSO) regression, extreme gradient boosting (XGBoost), and a simplified nomogram-based scoring system were applied to differentiate IBD from non-IBD conditions.

**Results:**

Significant differences were observed across all clinical and metabolism-related variables (P < 0.001). IBD patients exhibited elevated inflammatory-metabolic-related biomarkers and dense inflammation-driven metabolic correlation networks, whereas IBS patients showed metabolic profiles similar to healthy controls. XGBoost achieved the best diagnostic performance (AUC = 0.992), followed by LASSO (AUC = 0.978). A simplified scoring system incorporating sex, age, log-transformed fecal calprotectin (Log^FC^), log-transformed C-reactive protein (Log^CRP^), hemoglobin (HB), albumin (ALB), white blood cell count (WBC) and platelet count (PLT) achieved an AUC of 0.910, outperforming fecal calprotectin (FC) alone (AUC = 0.844) and the FC-CRP combination (AUC = 0.855). The scoring system demonstrated substantial clinical net benefit and correlated with colonoscopic inflammation severity (AUC = 0.761). Inflammatory and metabolism-related biomarkers were the strongest predictors of IBD.

**Conclusion:**

IBD is characterized by distinct multidimensional metabolism-related biomarker signatures. The proposed machine learning-based scoring system integrates these metabolism-related biomarkers into a reliable, non-invasive tool for invasive examination triage, potentially reducing unnecessary colonoscopies and improving clinical resource utilization.

## 1 Introduction

Inflammatory bowel disease (IBD) and irritable bowel syndrome (IBS) are among the most prevalent gastrointestinal disorders worldwide and represent a substantial and growing healthcare burden. In recent decades, the incidence and prevalence of IBD have increased rapidly in newly industrialized regions, particularly across Asia and Latin America, highlighting its emergence as a global public health challenge (Caron et al., 2024; Ng et al., 2017). Crohn’s disease (CD) and ulcerative colitis (UC), the two principal subtypes of IBD, are characterized by chronic relapsing intestinal inflammation arising from complex interactions among genetic susceptibility, environmental exposures, gut microbiota, and immune dysregulation. In contrast, IBS is a functional gastrointestinal disorder characterized by recurrent abdominal symptoms in the absence of established intestinal inflammation. Despite their distinct pathophysiological mechanisms and clinical management strategies, IBD and IBS frequently present with overlapping gastrointestinal symptoms, creating substantial diagnostic challenges in routine clinical practice.

Colonoscopy combined with histopathological examination remains the diagnostic gold standard for intestinal diseases (Annese et al., 2013). However, colonoscopy is invasive, costly, and associated with patient discomfort and procedural risks. In addition, the requirements for bowel preparation and limited patient acceptance may further reduce adherence. Consequently, some patients may undergo invasive examinations that could potentially be deferred because currently available non-invasive approaches do not always provide sufficient accuracy for distinguishing inflammatory from non-inflammatory intestinal disorders. Therefore, accurate and accessible pre-endoscopic risk stratification tools are needed to identify patients who are more likely to benefit from timely colonoscopic evaluation.

Fecal calprotectin (FC) is one of the most widely used non-invasive biomarkers for the diagnosis and monitoring of IBD. Nevertheless, its diagnostic performance may be limited in specific clinical scenarios, particularly in patients with mild disease activity or isolated small-bowel involvement (Gacesa et al., 2021). Conventional inflammatory biomarkers, including C-reactive protein (CRP), erythrocyte sedimentation rate (ESR), serum amyloid A (SAA), and fecal occult blood (FOB), are frequently used as adjunctive indicators but generally provide limited diagnostic value when evaluated individually (Kim et al., 2017; Sakurai and Saruta, 2022; Liu et al., 2022; Sands, 2015). Increasing evidence indicates that intestinal inflammation is not confined to the gastrointestinal tract but is accompanied by systemic alterations involving nutritional status, hepatic function, renal homeostasis, and immune-related biological processes. These interconnected responses cannot be adequately captured by a single biomarker and may provide complementary information for disease characterization.

Recent advances in the understanding of the gut–organ axis have further highlighted the close relationship between intestinal inflammation and systemic physiological processes. The emerging concept of the gut–liver–kidney axis suggests that alterations in intestinal barrier integrity, immune activation, and microbiota-derived metabolites may influence extraintestinal biological functions and contribute to disease progression (Lin et al., 2025). Accordingly, routinely available laboratory parameters reflecting inflammatory activity, nutritional and protein status, hepatic function, and renal homeostasis may collectively provide clinically accessible indicators of systemic alterations associated with intestinal inflammation. In this context, the present study uses the term “metabolism-related biomarkers” to refer to routinely available clinical laboratory parameters that indirectly reflect systemic metabolic alterations associated with intestinal inflammation, rather than direct metabolites or metabolomic measurements. Their integrated patterns are therefore referred to as “metabolism-related biomarker signatures.”

Machine learning algorithms provide a powerful framework for integrating heterogeneous clinical and biological variables and identifying complex or nonlinear relationships among multiple predictors. Compared with single-biomarker approaches or conventional statistical models based on a limited number of predefined variables, machine learning methods may better capture multidimensional disease characteristics and potentially improve diagnostic discrimination. Recent studies have demonstrated the potential of artificial intelligence for the non-invasive assessment of intestinal inflammation and disease activity (Zhu et al., 2025; Lee et al., 2024; Dranga et al., 2024; Polat et al., 2022). However, most existing investigations have focused on individual biomarkers or disease activity prediction, whereas relatively few studies have explored the integration of multidimensional, routinely available metabolism-related biomarkers for pre-endoscopic IBD risk stratification.

Therefore, the present study aimed to characterize metabolism-related biomarker patterns across healthy controls, IBS patients, and patients with IBD and to develop an interpretable machine learning-based scoring system integrating multidimensional metabolism-related biomarkers. We hypothesized that intestinal inflammation is associated with coordinated alterations across multiple systemic biological domains, including inflammatory activity, metabolism, nutritional and protein status, hepatic function, and renal homeostasis. By integrating these complementary biomarkers, we sought to develop a clinically applicable tool for pre-endoscopic risk stratification and triage of patients with suspected IBD, with the potential to support timely referral for colonoscopy and more efficient allocation of endoscopic resources.

## 2 Materials and methods

### 2.1 Sample size

The sample size was determined based on the statistical requirements for multi-group comparison, binary diagnostic model construction and machine learning model training/validation, with a two-sided α of 0.05 and a power of 80%. A total of 729 participants were enrolled, including 313 healthy controls, 210 IBS, 100 UC and 106 CD patients, with a balanced ratio of non-IBD to IBD cases (2.5:1) to avoid sampling bias. The cohort was randomly split into a training set (70%, n = 511) for model development using metabolic biomarker variables and hyperparameter tuning, and an independent test set (30%, n = 218) for validation, which met the sample size demand of multivariate logistic regression (sample size ≥50×number of variables) and ensured the statistical validity of all analyses.

### 2.2 Study participants

A retrospective single-center study was conducted at the Fourth Affiliated Hospital of Zhejiang University School of Medicine. All enrolled patients were treated in the Outpatient and Emergency Department of Gastroenterology from July 2021 to November 2025, and the healthy controls underwent routine physical examinations at our center during the same period. This study was approved by the Institutional Ethics Committee of the Fourth Affiliated Hospital of Zhejiang University School of Medicine, No. K2026070. Diagnoses of UC and CD were confirmed in accordance with the clinical practice guidelines of the European Crohn’s and Colitis Organisation (ECCO) (Gordon et al., 2024), and IBS was diagnosed based on the Rome IV criteria (Drossman, 2016).

It is important to clarify that while a definitive diagnosis of IBS per the Rome IV criteria does not mandate colonoscopy, all patients with IBS and healthy controls in this cohort underwent colonoscopy for one of the following clinically indicated reasons: (1) To exclude organic diseases due to the presence of alarm symptoms (e.g., unintended weight loss, nocturnal symptoms or family history of colorectal cancer) as per the standard of care at our institution during the study period; (2) As part of a routine health screening program that included colonoscopy. All enrolled patients with IBS and healthy controls had macroscopically and histologically normal colonic mucosa, thereby confirming the absence of organic intestinal disease. This approach, while potentially introducing selection bias, ensures the diagnostic certainty of the non-IBD group against which our models were validated.

Inclusion criteria: (1) Patients with confirmed diagnosis of UC, CD or IBS; healthy controls with no gastrointestinal symptoms or abnormal physical examination findings; (2) Completion of full colonoscopy and pathological biopsy; (3) Availability of complete clinical, laboratory and follow-up data; and (4) Normal laboratory test results for healthy controls.

Exclusion criteria: (1) Comorbidity with other organic gastrointestinal diseases, severe liver/renal dysfunction, autoimmune diseases, malignant tumors or hematological disorders; (2) Receipt of immunosuppressive therapy, glucocorticoid therapy or intestinal surgery within 3 months prior to enrollment; (3) Pregnant or lactating women.

For model development, UC and CD were combined into a single IBD category because the primary clinical objective was to identify patients with suspected IBD who may require further endoscopic evaluation rather than to differentiate UC from CD.

### 2.3 Data collection

General clinical information and metabolism-related laboratory data were extracted from the electronic medical record system of the hospital. Clinical variables included demographic characteristics [sex, age, body mass index (BMI)], lifestyle factors (smoking and alcohol consumption), symptom duration, family history, past medical history and *Helicobacter pylori* infection status.

To comprehensively characterize disease-associated metabolic alterations, laboratory variables were categorized into four major biological domains: intestinal inflammatory metabolism, nutritional metabolism, hepatic metabolic function and renal metabolic homeostasis. The intestinal inflammatory metabolism panel included FOB, fecal white blood cell count (FWBC), FC, CRP, SAA, procalcitonin (PCT) and ESR. Nutritional metabolism indicators included HB, ALB, globulin (GLB) and the albumin-to-globulin ratio (A/G). Hepatic metabolism-related indices included alanine aminotransferase (ALT), aspartate aminotransferase (AST) and the AST/ALT ratio. Renal metabolic homeostasis indicators included urea nitrogen (UN), creatinine (Cr), uric acid (UA) and estimated glomerular filtration rate (eGFR). Tumor-associated metabolic-related biomarkers, including carcinoembryonic antigen (CEA), carbohydrate antigen 19-9 (CA19-9), carbohydrate antigen 72-4 (CA72-4) and carbohydrate antigen 24-2 (CA24-2), were also collected.

Collectively, these routinely available laboratory parameters were classified as metabolism-related biomarkers because they reflect different dimensions of systemic metabolic responses to intestinal inflammation rather than serving solely as organ-specific laboratory measurements. Specifically, inflammatory biomarkers (e.g., FC, CRP, ESR and SAA) represent inflammation-associated metabolic activity; hemoglobin and albumin reflect nutritional and protein metabolism; liver function indices characterize hepatic metabolic function and renal function indices indicate metabolic homeostasis. Together, these biomarkers were considered to represent multidimensional host metabolism-related biomarker signatures associated with intestinal diseases.

### 2.4 Statistical and bioinformatic analysis

All statistical analyses were performed using R software (version 4.5.0) and Python (version 3.10). Continuous variables with normal distributions are presented as mean ± standard deviation (SD) and were compared using one-way analysis of variance (ANOVA). Non-normally distributed variables are expressed as median and interquartile range [M (IQR)] and were compared using the Kruskal–Wallis test. Categorical variables are presented as frequencies and percentages and were compared using the chi-square test or Fisher’s exact test when appropriate. A two-sided P value <0.05 was considered statistically significant.

Because intestinal diseases may involve coordinated alterations across multiple biological systems, statistical analyses were performed not only to evaluate individual biomarkers but also to characterize disease-associated metabolism-related biomarker signatures. All laboratory and clinical variables were therefore analyzed within an integrated metabolic framework.

Spearman correlation analysis was performed to investigate interactions among clinical variables and metabolism-related biomarkers within each study group. Spearman correlation coefficients were selected because of their robustness to non-normal distributions and their ability to capture monotonic relationships between variables.

To identify the dominant components of disease-associated metabolism-related biomarker signatures, the 15 variables with the highest mean absolute correlation coefficients were selected for network visualization. Correlation heatmaps were subsequently generated to illustrate diagnosis-specific metabolic interaction patterns. Network centrality was quantified using the mean absolute Spearman correlation coefficient (mean |r|), reflecting the relative importance of individual biomarkers within the metabolic network.

These analyses were designed to characterize metabolic network remodeling across healthy controls, IBS patients and IBD patients and to identify the biological pathways most strongly associated with disease status.

### 2.5 Model construction and validation

Before model development, candidate variables were screened according to the predefined analytical strategy. Missing values were not universally excluded from the dataset. Owing to the native missing-value handling capability of XGBoost, samples with missing laboratory parameters were retained for XGBoost modelling. By contrast, complete-case analysis was applied for the LASSO-logistic regression, and samples containing missing values were excluded only for this model. Continuous variables were standardized before model development where required. Feature selection for the LASSO model was performed within the training cohort to avoid information leakage. For XGBoost, model hyperparameters were optimized using five-fold cross-validation within the training cohort, while the independent test cohort was reserved for final model evaluation. Model discrimination was assessed using the area under the receiver operating characteristic curve (AUC), and clinical utility was evaluated using decision curve analysis.

The entire cohort was randomly divided into a training set (70%, n = 511) and an independent test set (30%, n = 218) using a fixed random seed. The training set was used for feature selection, model development and hyperparameter optimization, whereas the independent test set was reserved exclusively for final model evaluation.

Five diagnostic approaches based on different metabolism-related biomarker strategies were developed to distinguish IBD (UC + CD) from non-IBD conditions (healthy controls + IBS): FC alone; FC-CRP combination; Least absolute shrinkage and selection operator (LASSO) regression; Extreme gradient boosting (XGBoost); Simplified nomogram-based scoring system.

LASSO regression was performed using the lambda.1se criterion to identify a parsimonious subset of metabolism-related predictors while minimizing overfitting and multicollinearity. XGBoost models were constructed using the full set of clinical and metabolism-related variables. To further reduce model overfitting, model hyperparameters were optimized using five-fold cross-validation within the training cohort. The independent test cohort was not involved in feature selection or hyperparameter tuning and was reserved exclusively for external performance evaluation.

To facilitate clinical implementation, a simplified scoring system was developed using a two-stage strategy. First, LASSO regression identified candidate biomarkers associated with disease-specific metabolism-related biomarker signatures. Subsequently, variables were refined according to clinical availability, objectivity and routine applicability.

This process yielded eight readily obtainable metabolism-related predictors, including sex, age, log-transformed FC, log-transformed CRP, HB, ALB, WBC and PLT. These biomarkers collectively represent multiple dimensions of host metabolism, including inflammatory metabolism, nutritional metabolism and systemic metabolic homeostasis. ROC curves were compared using paired DeLong tests because all models were evaluated on the same independent test cohort. Holm correction was applied to adjust for multiple pairwise comparisons among different models. A two-sided adjusted P value < 0.05 was considered statistically significant.

A multivariable logistic regression model was then constructed and visualized as a nomogram. Weighted scores were assigned according to regression coefficients, and total scores were converted into individualized probabilities of IBD.

**FIGURE 1:**
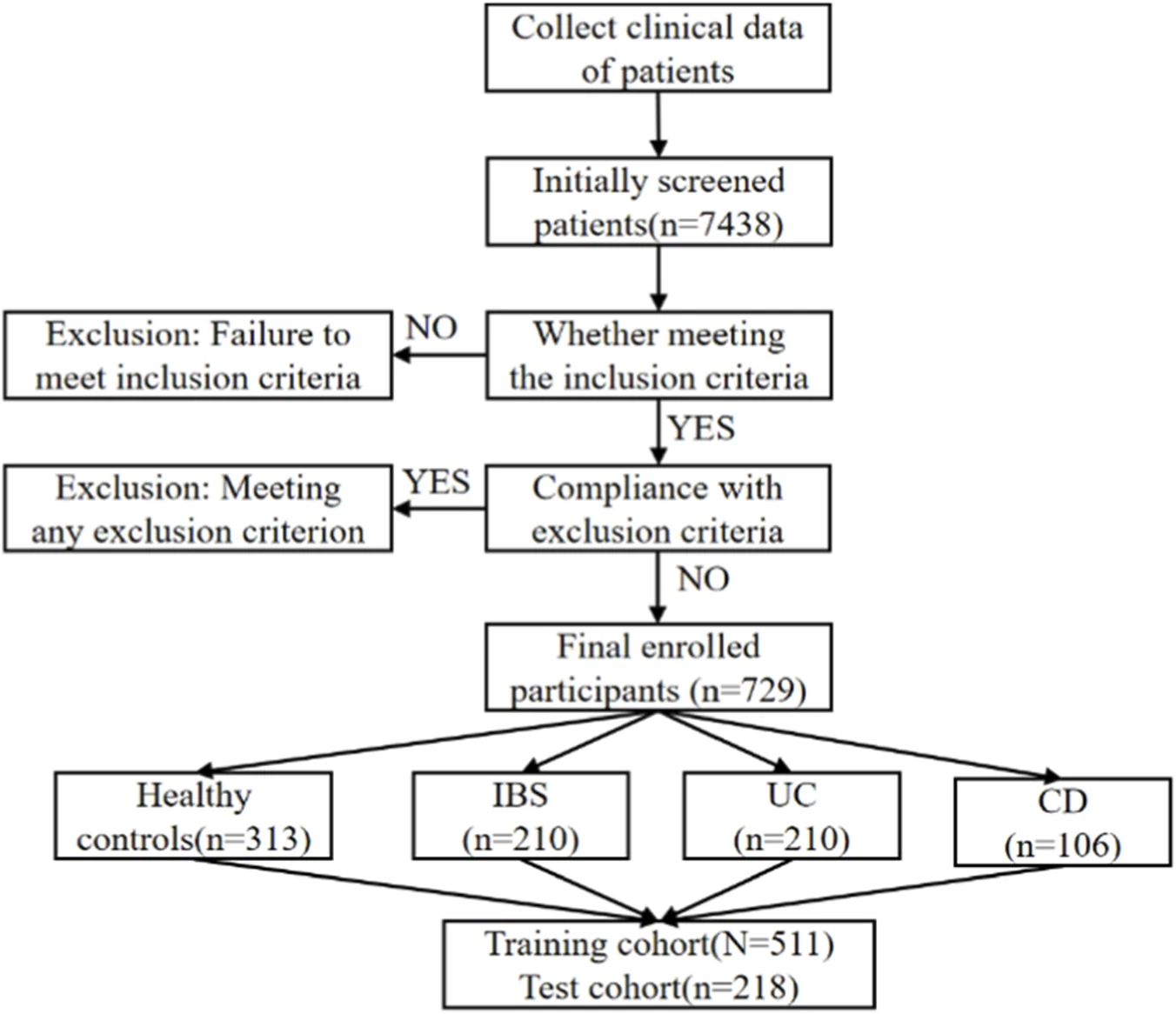
Patient selection flowchart.

Conceptually, the study framework was based on the hypothesis that chronic intestinal inflammation induces multidimensional metabolic perturbations involving inflammatory activity, nutritional metabolism, hepatic metabolism and renal metabolic homeostasis. Machine learning algorithms were subsequently applied to integrate these metabolism-related biomarker signatures for invasive examination triage. A flowchart illustrating participant enrollment and study selection has been added as Figure 1.

## 3 Results

### 3.1 Demographic, clinical and metabolic characteristics of the study population

A total of 729 participants were included in the study, comprising 313 healthy controls, 210 patients with IBS, 100 patients with UC and 106 patients with CD. Significant differences were observed among the four groups across demographic characteristics, clinical features and metabolism-related laboratory biomarkers (Table 1).

**TABLE 1:** Demographic, lifestyle and pathological characteristics of the study population [n (%)/mean ± SD/M (IQR)].

Variable	Healthy controls	IBS	UC	CD
Sample size	313	210	100	106
General clinical data				
Demographic characteristics				
Sex (male), n (%)	89 (28.43)	59 (28.10)	39 (39.00)	39 (36.79)
Age, mean ± SD	44.38 ± 9.60	34.96 ± 13.47	45.93 ± 15.45	34.16 ± 12.93
BMI, M (IQR)	24.30 (4.42)	23.20 (4.63)	21.80 (3.70)	20.06 (5.65)
Lifestyle factors				
Smoking habit(Y), n (%)	52 (16.61)	4 (1.90)	19 (19.00)	16 (15.09)
Alcohol habit(Y), n (%)	41 (13.10)	9 (4.29)	18 (18.00)	5 (4.72)
Clinical symptoms				
Duration of symptom, days, M (IQR)	NA	90 (344)	365 (1,065)	730 (1,665)
Medical history				
Family history(Y), n (%)	130 (41.53)	178 (84.76)	17 (17.00)	9 (8.49)
Past medical history(Y), n (%)	98 (31.31)	19 (9.05)	34 (34.00)	40 (37.74)
*H. pylori* infection(Y), n (%)	19 (6.07)	15 (7.14)	4 (4.00)	6 (5.66)
Pathological/Colonoscopic diagnosis				
Normal, n (%)	313 (100.00)	157 (74.76)	19 (19.00)	2 (1.89)
Non-neoplastic lesion, n (%)	NA	45 (21.43)	78 (78.00)	104 (98.11)
Neoplastic lesion, n (%)	NA	8 (3.81)	3 (3.00)	NA
Laboratory indices				
Fecal routine				
Fecal occult blood				
Negative, n (%)	296 (94.57)	183 (87.14)	38 (38.00)	76 (71.70)
1+, n (%)	13 (4.15)	15 (7.14)	28 (28.00)	11 (10.38)
2+, n (%)	4 (1.28)	5 (2.38)	17 (17.00)	10 (9.43)
3+, n (%)	NA	7 (3.33)	14 (14.00)	8 (7.55)
4+, n (%)	NA	NA	3 (3.00)	1 (0.94)
Fecal WBC				
Negative, n (%)	311 (99.36)	199 (94.76)	58 (58.00)	97 (91.51)
0–3, n (%)	2 (0.64)	4 (1.90)	4 (4.00)	2 (1.89)
3–6, n (%)	NA	2 (0.95)	5 (5.00)	1 (0.94)
6–10, n (%)	NA	2 (0.95)	6 (6.00)	NA
10–20, n (%)	NA	1 (0.48)	3 (3.00)	2 (1.89)
20–40, n (%)	NA	1 (0.48)	7 (7.00)	2 (1.89)
40–100, n (%)	NA	1 (0.48)	10 (10.00)	NA
>100, n (%)	NA	NA	7 (7.00)	2 (1.89)
Blood routine				
WBC, *10^9^/L, M (IQR)	6.10 (2.05)	NA	6.45 (2.95)	5.75 (2.13)
PLT, *10^9^/L, M (IQR)	229.00 (62.00)	NA	247.50 (123.75)	246.00 (110.00)
HB, g/L, M (IQR)	151.00 (23.50)	NA	130.00 (36.00)	133.50 (32.25)
Tumor markers				
CEA, ng/mL, M (IQR)	1.71 (1.26)	2.02 (1.39)	1.82 (1.43)	0.00 (1.15)
CA199, U/mL, M (IQR)	6.11 (8.71)	5.91 (5.33)	0.00 (6.06)	0.00 (3.93)
CA724, U/mL, M (IQR)	1.45 (2.21)	NA	1.38 (1.64)	1.28 (1.51)
CA242, U/mL, M (IQR)	5.96 (7.28)	NA	6.89 (6.54)	0.00 (3.74)
Inflammatory indicators				
FC, μg/g, M (IQR)	16.20 (19.30)	16.80 (22.38)	124.00 (249.90)	128.55 (400.60)
CRP, mg/L, M (IQR)	1.10 (1.70)	NA	2.10 (16.2)	2.65 (12.85)
SAA, mg/L, M (IQR)	NA	1.00 (13.95)	3.60 (33.13)	1.50 (44.85)
PCT, ng/mL, M (IQR)	NA	NA	0.058 (0.65)	0.073 (0.15)
ESR, mm/h, M (IQR)	0.00 (3.00)	2.00 (1.00)	12.00 (22.00)	6.00 (18.00)
Liver function tests				
ALB, g/L, M (IQR)	44.90 (3.40)	45.80 (1.25)	41.00 (5.15)	40.40 (6.32)
GLB, g/L, M (IQR)	27.80 (4.20)	26.70 (3.45)	28.20 (6.15)	27.70 (7.55)
ALB/GLB, M (IQR)	1.60 (0.20)	1.70 (0.20)	1.40 (0.30)	1.40 (0.43)
AST, U/L, M (IQR)	21.00 (7.00)	20.00 (12.50)	19.00 (7.00)	18.00 (7.00)
ALT, U/L, M (IQR)	23.00 (19.00)	20.00 (15.25)	16.00 (11.00)	13.00 (10.00)
AST/ALT, M (IQR)	1.00 (0.50)	1.00 (0.45)	1.20 (0.70)	1.30 (0.60)
Renal function tests				
Urea, mmol/L, M (IQR)	5.01 (1.68)	4.98 (1.21)	4.31 (1.89)	4.44 (2.09)
Cr, μmol/L, M (IQR)	68.00 (20.00)	71.00 (13.00)	68.50 (21.00)	66.00 (23.50)
UA, μmol/L, M (IQR)	345.40 (126.65)	379.00 (104.15)	302.00 (93.05)	324.75 (104.40)
eGFR, mL/min/1.73m^2^, M (IQR)	111.00 (28.50)	117.00 (29.50)	98.50 (111.75)	118.00 (40.25)

NA indicates that no corresponding data were available.

Compared with healthy controls and IBS patients, individuals with IBD exhibited a distinct pattern of multidimensional metabolic alterations. Specifically, UC and CD patients demonstrated significantly elevated intestinal inflammatory metabolism markers, including FC, CRP, SAA, ESR and FOB positivity, indicating a substantially greater inflammatory burden. In parallel, markers reflecting nutritional metabolism were markedly altered, with significantly lower HB and ALB levels observed in both UC and CD groups.

In addition, IBD patients displayed characteristic changes in hepatic metabolism-related indices, including reduced ALT levels and increased AST/ALT ratios, together with alterations in renal metabolic homeostasis reflected by lower UA concentrations and reduced eGFR values. These findings suggest that the biological consequences of intestinal inflammation extend beyond the gastrointestinal tract and involve systemic metabolic perturbations affecting multiple organ systems.

In contrast, the metabolic profiles of IBS patients closely resembled those of healthy controls across most laboratory variables, supporting the concept that IBS is primarily a functional gastrointestinal disorder without substantial systemic metabolic remodeling.

### 3.2 Diagnosis-specific metabolic network remodeling

**FIGURE 2:**
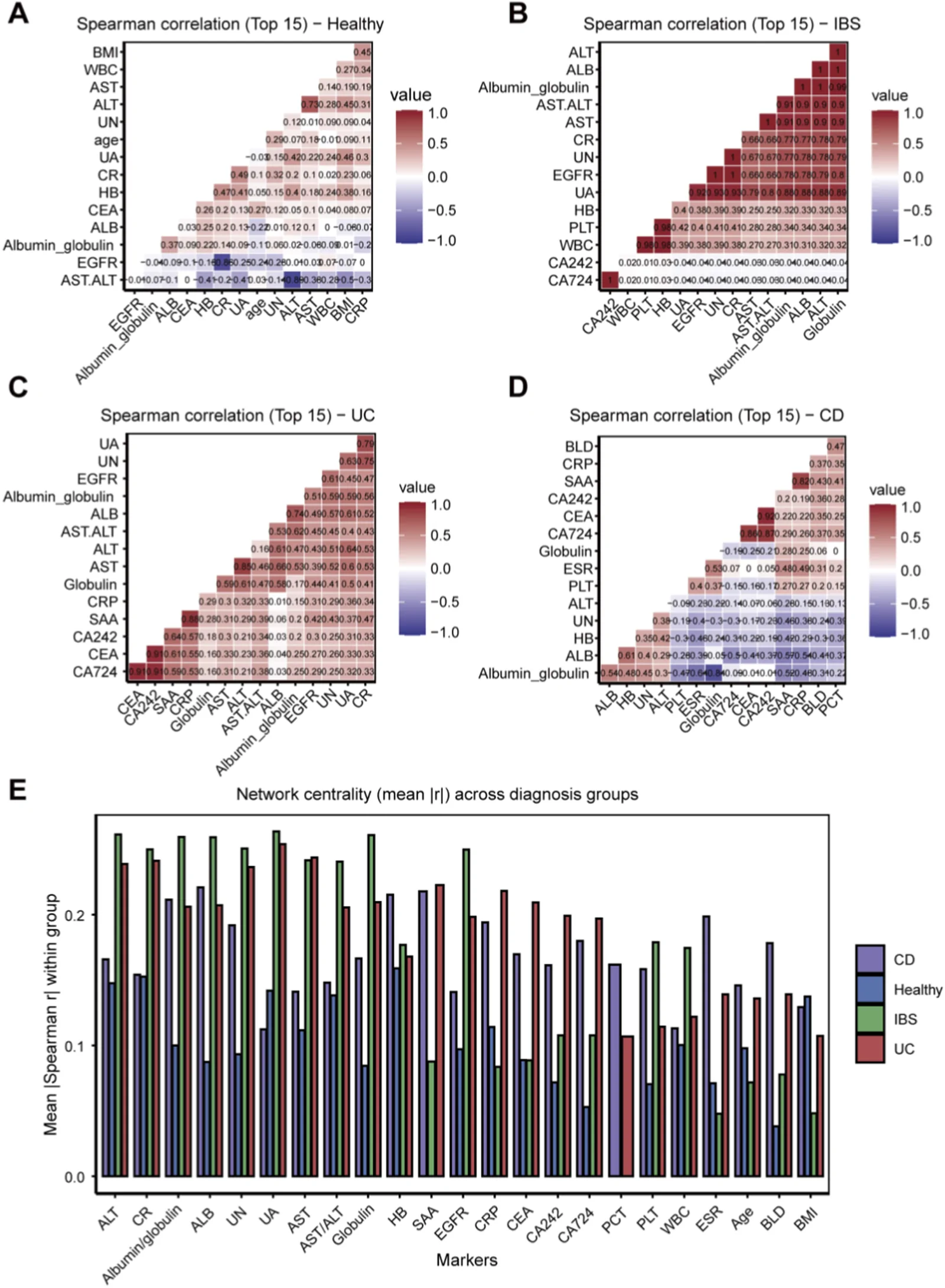
Diagnosis-specific metabolic network remodeling and network centrality analysis **(A-D)** Spearman correlation heatmaps illustrating diagnosis-specific metabolic interaction patterns in healthy controls, irritable bowel syndrome (IBS), ulcerative colitis (UC) and Crohn’s disease (CD), respectively. The top 15 variables with the highest mean absolute correlation coefficients were selected. Red indicates positive correlations and blue indicates negative correlations, with color intensity corresponding to correlation strength **(E)** Network centrality analysis showing the mean absolute Spearman correlation coefficient (mean |r|) of key metabolism-related biomarkers within each diagnostic group. Increased centrality indicates a greater contribution of the corresponding biomarker to the overall metabolic network. The progressive transition from healthy controls and IBS to UC and CD demonstrates substantial inflammation-driven metabolic network remodeling involving inflammatory metabolism, nutritional metabolism, hepatic metabolic function and renal metabolic homeostasis.

To explore disease-associated interactions among metabolism-related biomarkers, diagnosis-specific Spearman correlation networks were constructed for healthy controls, IBS, UC and CD groups (Figures 2A–D).

Healthy controls exhibited relatively stable metabolic interaction patterns characterized by balanced correlations among nutritional, hepatic and renal metabolism-related biomarkers. Similar network structures were observed in IBS patients, indicating preservation of overall metabolic homeostasis despite the presence of gastrointestinal symptoms.

In contrast, profound metabolic network remodeling was observed in both UC and CD. Correlation networks became substantially denser and were characterized by strong interactions linking inflammatory metabolism markers with nutritional, hepatic and renal metabolism-related biomarkers. FC, CRP, ESR and SAA emerged as highly interconnected nodes, suggesting that inflammatory activity serves as a central driver of disease-associated metabolic perturbations.

Network centrality analysis further demonstrated a progressive increase in the importance of inflammation-related biomarkers from healthy controls and IBS to UC and CD (Figure 2E). These findings indicate that chronic intestinal inflammation is accompanied by coordinated alterations across multiple metabolic systems rather than isolated changes in individual laboratory variables.

Collectively, the network analyses revealed that IBD is characterized by an inflammation-driven metabolic signature involving extensive interactions among inflammatory metabolism, nutritional metabolism, hepatic metabolic function and renal metabolic homeostasis.

### 3.3 Development of machine learning models based on metabolism-related biomarker signatures

**FIGURE 3:**
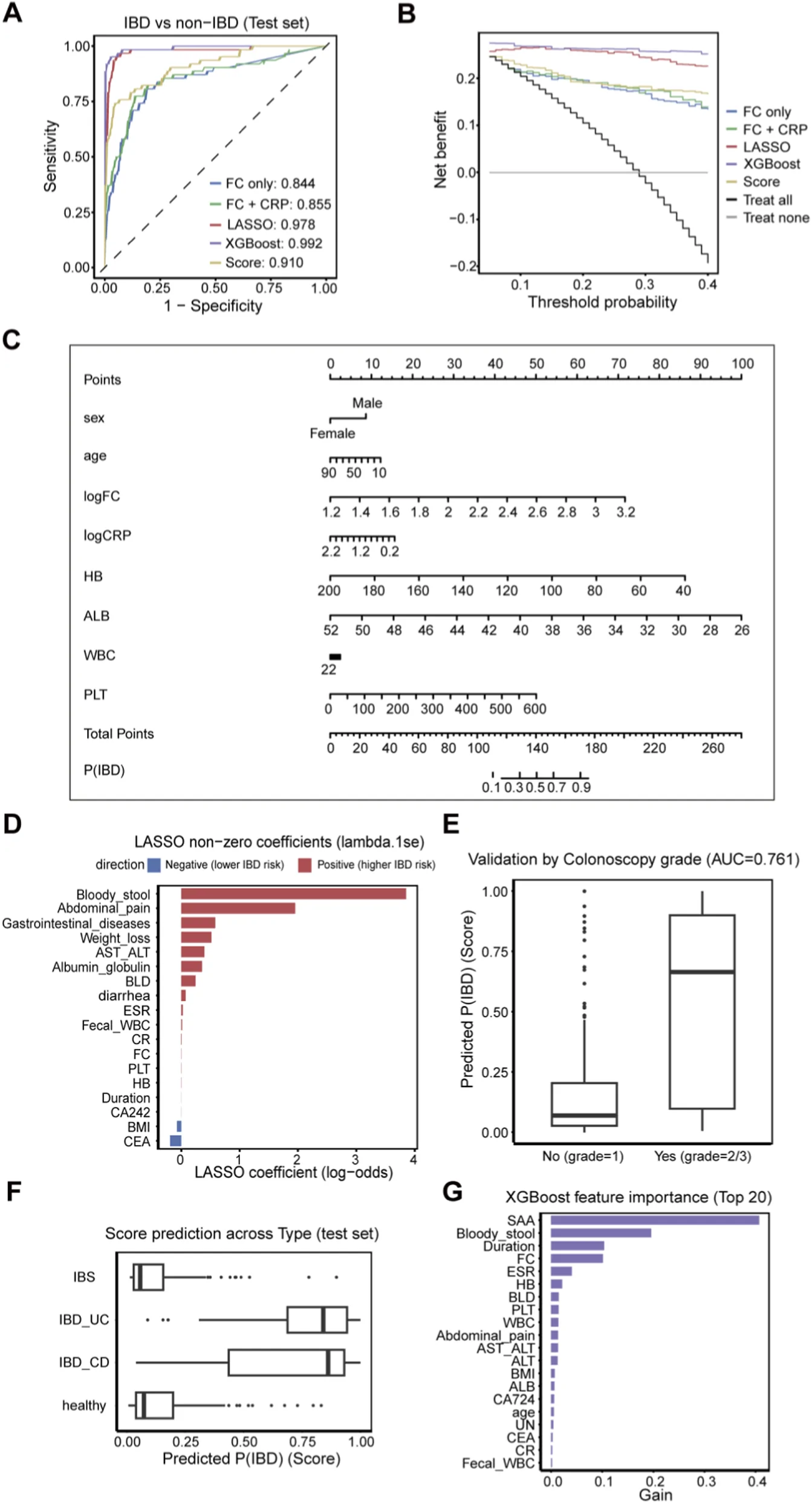
Diagnostic performance and clinical validation of machine learning models integrating multidimensional metabolism-related biomarker signatures **(A)** Receiver operating characteristic (ROC) curves comparing five diagnostic approaches for differentiating inflammatory bowel disease (IBD) from non-IBD conditions in the independent test set **(B)** Decision curve analysis (DCA) demonstrating the clinical net benefit of each diagnostic model across different threshold probabilities **(C)** Nomogram of the simplified metabolism-based scoring system for estimating the probability of IBD **(D)** Regression coefficients of predictors retained by least absolute shrinkage and selection operator (LASSO) regression at lambda.1se **(E)** ROC curve evaluating the ability of the simplified scoring system to predict moderate-to-severe endoscopic inflammation **(F)** Distribution of predicted probabilities generated by the simplified scoring system across healthy controls, IBS, UC and CD groups **(G)** Feature importance ranking of the XGBoost model. Higher scores indicate greater contributions of individual metabolism-related biomarkers to diagnostic prediction. The figure demonstrates that machine learning algorithms effectively integrate multidimensional metabolism-related biomarker signatures and provide superior diagnostic performance compared with conventional biomarker-based approaches.

To determine whether multidimensional metabolism-related biomarker signatures could improve diagnostic discrimination between IBD and non-IBD conditions, five diagnostic approaches were evaluated in the independent test cohort (Figure 3A).

Among the conventional biomarker-based methods, FC alone achieved an AUC of 0.844, while the FC-CRP combination yielded a modest improvement with an AUC of 0.855. Although both approaches demonstrated acceptable diagnostic performance, their ability to capture the complexity of disease-associated metabolic perturbations remained limited.

In contrast, machine learning models integrating multidimensional metabolism-related biomarkers achieved substantially superior diagnostic accuracy. The LASSO model achieved an AUC of 0.978, while the XGBoost model demonstrated near-perfect discrimination with an AUC of 0.992. Pairwise paired DeLong tests demonstrated that LASSO and XGBoost significantly outperformed both FC alone and FC plus CRP in the test set (all Holm-adjusted P < 0.001). XGBoost showed the highest numerical AUC, although its performance did not differ significantly from that of LASSO (adjusted P = 0.081). The Score model outperformed FC alone (adjusted P = 0.038), but not FC plus CRP (adjusted P = 0.068). These findings indicate that the diagnostic information contained within host metabolism-related biomarker signatures extends far beyond that provided by individual inflammatory biomarkers and can be effectively extracted through machine learning algorithms.

### 3.4 Construction of a clinically applicable metabolic scoring system

Although the XGBoost model achieved the highest predictive performance, its complexity may limit routine clinical implementation. Therefore, an interpretable nomogram-based scoring system was developed to facilitate bedside application (Figure 3C).

Using LASSO-based feature selection and clinical applicability criteria, eight predictors were ultimately retained, including sex, age, log-transformed FC, log-transformed CRP, HB, ALB, WBC and PLT (Figure 3D).

Notably, these variables collectively represent multiple dimensions of disease-associated metabolism, including intestinal inflammatory metabolism (FC, CRP, WBC, PLT), nutritional metabolism (HB, ALB) and host biological susceptibility (sex and age). The resulting scoring system therefore provides a simplified representation of the multidimensional metabolic signature associated with IBD.

In the independent test set, the scoring system achieved an AUC of 0.910, substantially outperforming FC alone and the FC-CRP combination while maintaining excellent interpretability and clinical applicability.

### 3.5 Clinical utility of the metabolic scoring system

Decision curve analysis demonstrated that the simplified scoring system provided greater net clinical benefit than conventional biomarker strategies across clinically relevant threshold probabilities and consistently outperformed conventional biomarker-based strategies (Figure 3B).

To further evaluate its clinical relevance, the scoring system was assessed for its ability to predict colonoscopic inflammation severity. Moderate-to-severe endoscopic inflammation was defined as a Mayo Endoscopic Score ≥2 for UC and a SES-CD score corresponding to moderate or severe disease for CD. The model achieved an AUC of 0.761 for identifying moderate-to-severe endoscopic inflammation (Figure 3E), indicating that the metabolic signature captured by the model reflects not only disease presence but also the biological severity of intestinal inflammation.

Furthermore, predicted probabilities differed significantly across healthy controls, IBS, UC and CD groups (Figure 3F). A clear gradient was observed, with the lowest scores in healthy controls, intermediate scores in IBS patients and substantially higher scores in UC and CD patients. This pattern further supports the biological validity of the identified metabolic signature.

### 3.6 Key metabolic determinants of IBD diagnosis

Feature importance analysis of the XGBoost model revealed that inflammation-related metabolic-related biomarkers were the dominant contributors to diagnostic prediction (Figure 3G).

Among all variables, FC, CRP, SAA, ALB, HB and PLT ranked among the most influential predictors. These biomarkers represent interconnected aspects of inflammatory metabolism, nutritional metabolism and systemic metabolic homeostasis, highlighting the multidimensional nature of disease-associated metabolic remodeling.

The predominance of these variables suggests that IBD diagnosis is driven not by isolated inflammatory abnormalities but rather by integrated perturbations across multiple biological and metabolic pathways. This observation provides a biologically plausible explanation for the superior diagnostic performance achieved by machine learning models integrating multidimensional metabolism-related biomarker signatures.

## 4 Discussion

The present study systematically characterized the demographic, clinical and metabolism-related biomarker profiles of healthy controls, IBS patients and IBD patients and developed a machine learning-based scoring system for invasive examination triage. Several important findings emerged from this study. First, IBD was associated with profound multidimensional metabolic alterations involving intestinal inflammatory metabolism, nutritional metabolism, hepatic metabolic function and renal metabolic homeostasis, whereas the metabolic profiles of IBS patients closely resembled those of healthy controls. Second, diagnosis-specific metabolic network analyses revealed substantial inflammation-driven metabolic remodeling in UC and CD. Third, machine learning models integrating multidimensional metabolism-related biomarker signatures achieved excellent diagnostic performance for distinguishing IBD from non-IBD conditions. Finally, a simplified nomogram-based scoring system incorporating eight routinely available biomarkers retained high diagnostic accuracy and demonstrated substantial clinical utility for endoscopic triage.

### 4.1 Multidimensional metabolic characteristics of IBD

A central finding of this study is that IBD should not be viewed solely as a localized inflammatory disorder of the intestine. Instead, chronic intestinal inflammation appears to induce widespread systemic metabolic perturbations involving multiple biological pathways. Consistent with previous studies, FC, CRP, SAA and ESR were markedly elevated in IBD patients, reflecting active intestinal and systemic inflammatory responses (Gacesa et al., 2021; Sakurai and Saruta, 2022; Liu et al., 2022; Sands, 2015). However, the metabolic alterations observed in our cohort extended beyond inflammatory biomarkers alone. Accordingly, the concept of metabolism-related biomarkers adopted in the present study extends beyond conventional metabolomic molecules. We propose that routinely available laboratory parameters reflecting inflammatory activity, nutritional status, hepatic function and renal homeostasis collectively characterize systemic metabolic remodeling associated with chronic intestinal inflammation. Although these biomarkers are not direct metabolites, they provide clinically accessible surrogates of disease-associated metabolic disturbances. Therefore they can be integrated into multidimensional metabolism-related biomarker signatures for machine learning analysis.

Patients with IBD exhibited significantly reduced HB and ALB levels, suggesting impaired nutritional metabolism and protein homeostasis. Chronic intestinal inflammation may contribute to anemia through multiple mechanisms, including impaired iron absorption, cytokine-mediated suppression of erythropoiesis and chronic blood loss. Similarly, hypoalbuminemia may result from reduced protein synthesis, increased intestinal protein loss and persistent inflammatory activity. Together, these findings indicate that nutritional metabolism constitutes an important component of the biological phenotype of IBD.

In addition, alterations were observed in hepatic metabolism-related indices and renal metabolic homeostasis indicators. Although these abnormalities were generally mild, they support the concept that chronic intestinal inflammation is associated with systemic metabolic consequences affecting multiple organs. These observations are consistent with accumulating evidence demonstrating that intestinal inflammation can influence distant metabolic pathways through immune, microbial and endocrine mechanisms.

### 4.2 The gut-liver-kidney metabolic axis and systemic metabolic remodeling

Recent advances in systems biology have highlighted the importance of inter-organ communication in chronic inflammatory diseases. The emerging concept of the gut–liver–kidney metabolic axis provides a plausible biological framework for interpreting the metabolic alterations observed in the present study (Lin et al., 2025).

Persistent intestinal inflammation may disrupt epithelial barrier integrity and alter gut microbial composition, resulting in increased translocation of microbial products, inflammatory mediators and metabolism-related signaling molecules into the systemic circulation. These factors can subsequently influence hepatic protein synthesis, lipid and amino acid metabolism, and renal metabolic regulation. Consequently, biomarkers such as ALB, AST, ALT, UA and eGFR may not simply represent organ-specific laboratory measurements but may also reflect systemic metabolic responses to chronic intestinal inflammation.

From this perspective, the observed metabolic abnormalities likely represent coordinated metabolic remodeling rather than isolated laboratory alterations. The coexistence of inflammatory activation, nutritional impairment and extraintestinal metabolic dysfunction further supports the concept that IBD is a systemic disease characterized by multidimensional metabolic perturbations.

### 4.3 Metabolic network remodeling in IBD

Another notable finding of this study is the identification of diagnosis-specific metabolic interaction networks. Compared with healthy controls and IBS patients, UC and CD exhibited substantially denser correlation networks characterized by strong interactions among inflammatory, nutritional, hepatic and renal metabolism-related biomarkers.

Network centrality analysis revealed that inflammation-related biomarkers occupied dominant positions within the metabolic networks of IBD patients. These findings suggest that inflammation-related biomarkers are closely associated with multiple biological systems and may reflect interconnected systemic responses to intestinal inflammation. Importantly, the network analyses indicate that disease-associated biomarkers should not be interpreted as independent variables but rather as interconnected components of a dynamic metabolic system.

The concept of metabolic network remodeling may help explain why single biomarkers often demonstrate limited diagnostic performance. Individual laboratory indicators capture only specific aspects of disease biology, whereas network-based approaches reflect the integrated consequences of multiple interacting pathways. Therefore, multidimensional metabolism-related biomarker signatures may provide a more comprehensive representation of disease status than isolated biomarkers.

### 4.4 Machine learning integration of metabolism-related biomarker signatures

The superior performance of the machine learning models observed in this study further supports the value of integrating multidimensional metabolic information. Traditional diagnostic approaches based on FC alone or FC combined with CRP achieved only moderate improvements in diagnostic accuracy. In contrast, both LASSO and XGBoost models achieved excellent discrimination between IBD and non-IBD conditions. Overall, the machine-learning models, particularly LASSO and XGBoost, demonstrated superior discrimination compared with conventional biomarker-based models, whereas no statistically significant difference was observed between LASSO and XGBoost.

A likely explanation for this improvement is the ability of machine learning algorithms to capture complex nonlinear interactions among multiple metabolism-related biomarkers. Unlike conventional statistical approaches that often evaluate variables independently, machine learning methods can identify higher-order relationships and hidden patterns within multidimensional datasets. Consequently, these algorithms are particularly well suited for extracting clinically relevant information from complex metabolism-related biomarker signatures.

Notably, the simplified scoring system retained excellent diagnostic performance despite incorporating only eight routinely available variables. Although XGBoost demonstrated the highest discrimination, the simplified scoring system showed a favorable balance between predictive performance and clinical interpretability. This finding suggests that a relatively small subset of carefully selected metabolism-related biomarkers can effectively capture the essential biological characteristics of IBD while maintaining interpretability and clinical practicality.

### 4.5 Clinical implications for invasive examination triage

The increasing prevalence of intestinal diseases has placed considerable pressure on endoscopic services worldwide. Although colonoscopy remains indispensable for definitive diagnosis, its invasive nature, cost and limited availability highlight the need for effective pre-endoscopic risk stratification tools.

Despite the lower discrimination compared with XGBoost, the simplified scoring system may provide greater clinical feasibility because of its transparency, simplicity, and reliance on routinely available laboratory tests. Unlike the “black-box” nature of XGBoost, the scoring system allows clinicians to directly interpret individual risk contributions and apply the model without specialized computational infrastructure. The simplified scoring system requires only routinely available laboratory tests, is fully interpretable and can be easily integrated into routine outpatient or primary care workflows. By integrating multidimensional metabolism-related biomarker signatures into a clinically interpretable framework, the model provides individualized estimates of IBD probability using routinely available laboratory tests. Patients with high predicted probabilities may be prioritized for timely colonoscopic evaluation, whereas those with low predicted probabilities may initially undergo non-invasive monitoring and follow-up.

Importantly, the model also demonstrated a significant association with endoscopic inflammation severity, suggesting that the identified metabolic signature reflects not only disease presence but also underlying inflammatory burden. Consequently, this approach may contribute to more efficient allocation of endoscopic resources and facilitate precision diagnostic decision-making in routine clinical practice. The intended clinical application is the initial evaluation of patients presenting with chronic diarrhea, abdominal pain, or suspected inflammatory bowel disease before colonoscopy. The scoring system is designed to support referral prioritization rather than establish a definitive diagnosis. It may facilitate more efficient allocation of endoscopic resources and improve clinical decision-making, particularly in resource-limited healthcare settings. But the scoring system is intended solely for pre-endoscopic risk stratification and referral prioritization. It should not be used as a stand-alone diagnostic tool or as a substitute for colonoscopy and histopathological examination.

### 4.6 Strengths and limitations

This study has several strengths. First, it included a relatively large and clinically diverse cohort comprising healthy controls, IBS patients, UC patients, and CD patients, enabling comprehensive comparisons across different intestinal disease states. Second, routinely available metabolism-related biomarkers were integrated into a unified analytical framework, allowing the characterization of disease-associated biomarker patterns and their interrelationships. Third, multiple complementary analytical approaches, including diagnostic performance evaluation, decision curve analysis, and correlation network analysis, were employed to assess model discrimination, potential clinical utility, and biomarker interactions.

Several limitations should also be acknowledged. First, this was a retrospective single-center study, and the patient characteristics, referral patterns, disease spectrum, and clinical practices of the study population may differ from those of other healthcare institutions. Therefore, the excellent predictive performance observed in the present cohort, particularly the high discrimination of the XGBoost model, should be interpreted cautiously and may not be directly generalizable to broader clinical populations. Prospective multicenter studies with independent external validation are warranted to confirm the robustness and generalizability of the proposed scoring system before routine clinical implementation.

Second, the inclusion of healthy controls and IBS patients as non-IBD populations may have introduced selection bias. Because all healthy controls and IBS patients underwent colonoscopy, these participants may not fully represent the broader non-IBD population encountered in routine primary care or general clinical practice. In addition, healthy controls and IBS patients differ substantially in their clinical characteristics and may contribute differently to model discrimination. Although the inclusion of IBS patients provided a clinically relevant symptomatic non-IBD population for comparison, the observed diagnostic performance may partly reflect differences between the selected study groups rather than performance across the full spectrum of real-world non-IBD populations.

Third, UC and CD were combined into a single IBD outcome for model development because the primary objective was to identify patients with suspected IBD who may require further endoscopic evaluation rather than to distinguish between UC and CD. Nevertheless, UC and CD have distinct pathophysiological characteristics, inflammatory patterns, and clinical manifestations, and this disease heterogeneity may influence model performance. Future studies with larger cohorts should evaluate whether disease-specific models provide additional diagnostic value.

Fourth, the metabolism-related biomarkers were measured at a single time point, precluding assessment of longitudinal changes associated with disease progression, treatment response, or changes in inflammatory activity. Longitudinal studies may provide further insight into the dynamic relationship between these biomarkers and disease status.

Fifth, although the selected biomarkers reflect multiple aspects of systemic metabolic responses, dedicated metabolomic measurements were not available in the present study. Therefore, the current findings should not be interpreted as a substitute for comprehensive systemic metabolic alterations. Future studies incorporating metabolomic and other multi-omics approaches may help further characterize the biological basis of the observed metabolism-related biomarker signatures and clarify the mechanisms underlying disease-associated metabolic alterations.

Finally, the proposed scoring system should be interpreted as a pre-endoscopic clinical triage and risk-stratification tool rather than a definitive diagnostic instrument. It is intended to assist clinical decision-making and patient prioritization for further evaluation, particularly in settings with limited endoscopic resources. It should not be considered a substitute for colonoscopy or histopathological examination, which remain essential for establishing a definitive diagnosis.

## 5 Conclusion

The proposed simplified scoring system integrates multidimensional metabolism-related biomarkers into an interpretable and clinically applicable tool for pre-endoscopic risk stratification. By identifying patients at increased risk of inflammatory bowel disease, it may facilitate more efficient allocation of endoscopic resources and support clinical decision-making. Nevertheless, this scoring system is intended solely as an adjunctive triage tool and should not be considered a substitute for colonoscopy or histopathological examination, which remain the gold standard for establishing a definitive diagnosis. Future prospective multicenter studies are warranted to further validate its clinical utility and generalizability.

## Data Availability

The raw data supporting the conclusions of this article will be made available by the authors, without undue reservation.
